# Ultrasound-guided arthroscopic communication enlargement surgery may be an ideal treatment option for popliteal cysts – indications and technique

**DOI:** 10.1186/s40634-020-00314-x

**Published:** 2020-11-30

**Authors:** Kazumi Goto, Isaku Saku

**Affiliations:** Department of Orthopaedic Surgery, Yaizu City Hospital, 1000, Dohbara, Yaizu-shi, Shizuoka 425-8505 Japan

**Keywords:** Popliteal cyst, Baker’s cyst, Arthroscopic surgery, Ultrasonography, Ultrasound-guided

## Abstract

**Purpose:**

Several studies have shown an excellent success rate of communication enlargement surgery for popliteal cysts (Baker’s cysts). Ultrasound-guided surgery can improve the accuracy of this procedure and may lead to better outcomes. This study describes a simple ultrasound-guided arthroscopic technique to manage popliteal cysts and reduce postoperative pain.

**Methods:**

After routine arthroscopic observation with a standard 2-portal approach, the arthroscope is redirected toward the posteromedial compartment from the anterolateral portal through the intercondylar notch. A posteromedial portal is then placed at this view. Subsequently, a contrast dye (indigo carmine) is injected into the popliteal cyst percutaneously using ultrasonography. This procedure makes it easier to find a capsular fold or valvular opening. The valvular opening between the semimembranosus and medial gastrocnemius is enlarged with a shaver and radiofrequency ablation. Cystectomy is not performed in any case. Finally, the irrigation fluid is suctioned, and the reduced cyst is visualized by ultrasound. Additionally, a periarticular multimodal drug injection is administered into the septum and inner wall of the cyst under ultrasound guidance.

**Conclusions:**

Ultrasound-guided arthroscopic surgery for popliteal cysts can ensure reproducibility and be effective for postoperative pain relief. Thus, this combined procedure may be an optimal treatment option.

## Background

Popliteal cysts are characterized by the enlargement of the gastrocnemius-semimembranosus bursa in the posteromedial region of the knee [13]. They were first reported by Adams in 1840 [1], and Baker described in 1877 that this synovial cyst communicates with the knee joint and is often associated with other intra-articular lesions [3]. Accordingly, popliteal cysts are also known as Baker’s cysts. Pathologically, it has been reported that the enlargement of the gastrocnemius-semimembranosus bursa is caused by increased joint fluid in communication channels. These channels have a valvular mechanism at the capsular fold on the posteromedial capsule, promoting a continuous unidirectional flow between the posterior joint capsule and bursa [21, 24, 25]. The prevalence of communication channels between the gastrocnemius-semimembranosus bursa and the knee joint cavity in adults varies, and previous studies have reported that a connection was observed in 30–71% of subjects [4, 11, 13, 14, 16]. Although various treatment options, including conservative treatment, open surgical resection, and arthroscopic surgery, have been reported, the optimal surgical treatment remains uncertain. The recurrence rate after a simple open resection reportedly ranges between 42% to 63% [6, 8, 10, 25]. If the valvular mechanism is not corrected during surgery, a continuous flow of joint fluid will occur. This may be why the recurrence rate after open surgery remains high in various studies. Therefore, the key to a successful surgery is the closure or enlargement of the communication between the cyst and the articular cavity [30].

In recent years, good results have been reported using arthroscopic resection of the communication, also known as communication enlargement surgery, with a recurrence rate of less than 10% [2, 7, 20, 23, 28]. At our institution, communication enlargement surgeries have been performed with ultrasound guidance, which is a more reliable and less invasive procedure. Nevertheless, no report has described the indications and surgical techniques employed in this procedure. Appropriate indications and surgical techniques are important to achieve satisfactory results. Therefore, this report describes the indications and surgical techniques for ultrasound-guided arthroscopic communication enlargement surgery.

### Indications for ultrasound-guided arthroscopic communication enlargement surgery

Ultrasound-guided arthroscopic surgery is commonly performed in symptomatic cases refractory to conservative treatment. For asymptomatic cases, we highly recommend supervised neglect; however, if the patient desires to undergo active treatment, the procedure can be performed, as described below. First, ultrasound-guided percutaneous treatment is performed, as described by Koroglu et al. [19]. Subsequently, after disinfection and local anesthesia induction, popliteal cysts are punctured using an 18G needle under ultrasonography. Ultrasound-guided puncture, aspiration, and corticosteroid injection are performed using a freehand technique by a well-experienced surgeon (K.G., 8 years of interventional ultrasonography experience) in our center. The cyst is almost completely decompressed, and 4 mg of Kenacort®^□^ (4 mg of triamcinolone acetonide) is subsequently administered into the cyst under ultrasound guidance. This treatment has a reported success rate of 87% and should be considered a first-line intervention since it is the most minimally invasive procedure [19, 29]. After 3 months of follow-up, surgical treatment is recommended in case of recurrence.

### Surgical technique

**Fig. 1:**
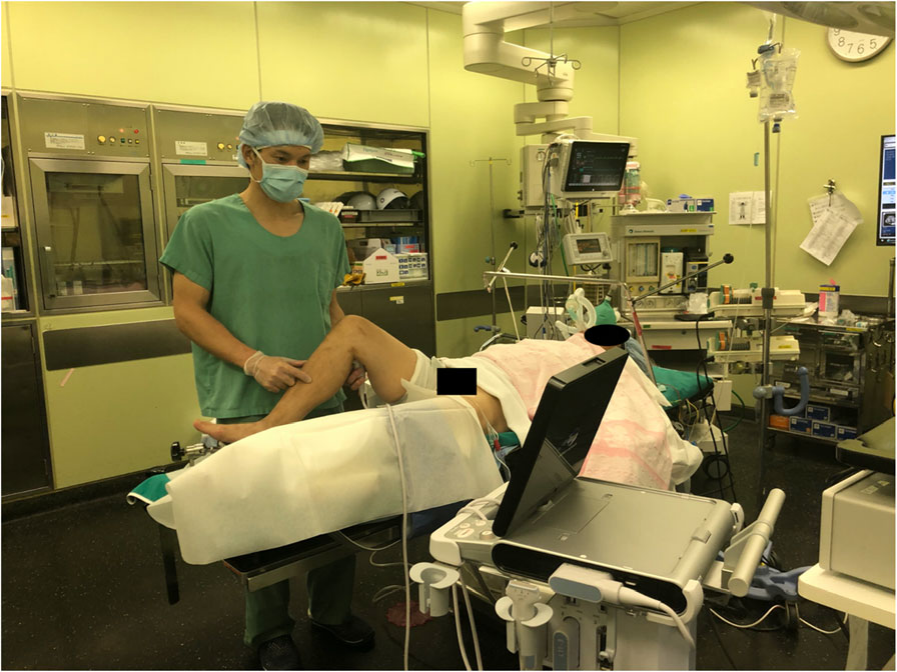
Preoperative setting and ultrasound evaluation in the operating room

**Fig. 2:**
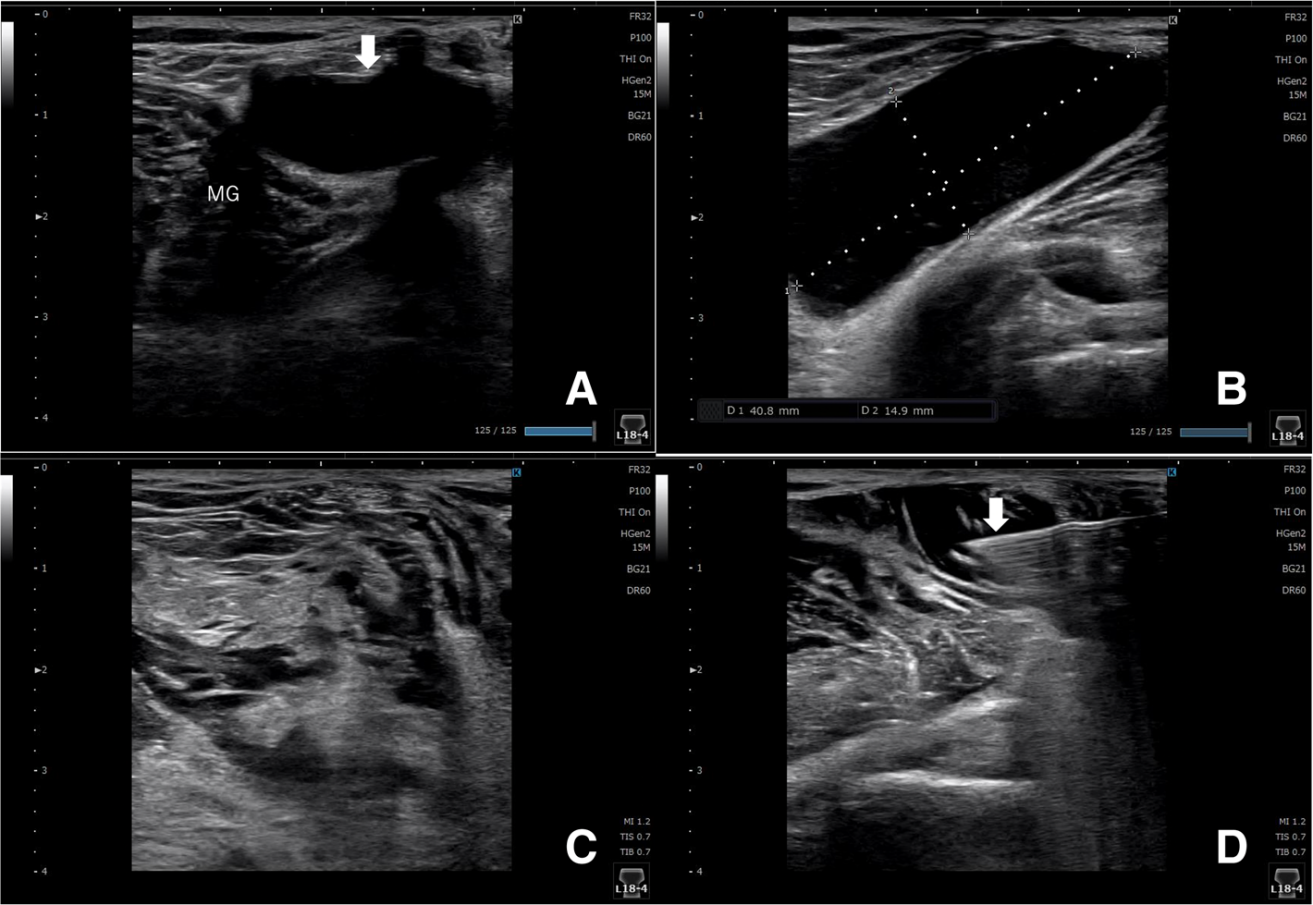
Ultrasound findings of the right knee before and after communication enlargement. **a** The short-axis view of the cyst (thin white arrow). **b** The long-axis view of the cyst and its size. **c** The size of the cyst is obviously reduced. **d** A periarticular multimodal drug is injected under ultrasonography (white arrow) to ensure postoperative pain relief. MG: medial gastrocnemius

Two Wakasugi body supports (Mizuho, Bunkyo-ku, Tokyo, Japan) are attached to the side rails so that 80° of knee flexion can be maintained. Increased flexion of more than 80° makes it difficult to obtain ultrasound imaging from the posterior knee, which may lead to poor visualization. In contrast, a decreased flexion of less than 80° makes arthroscopic manipulation difficult and provides a poor arthroscopic view. Ultrasonography is used to reconfirm the location and measure the size of the cyst before surgery (Fig. 1 and Fig. 2a-b).

**Fig. 3:**
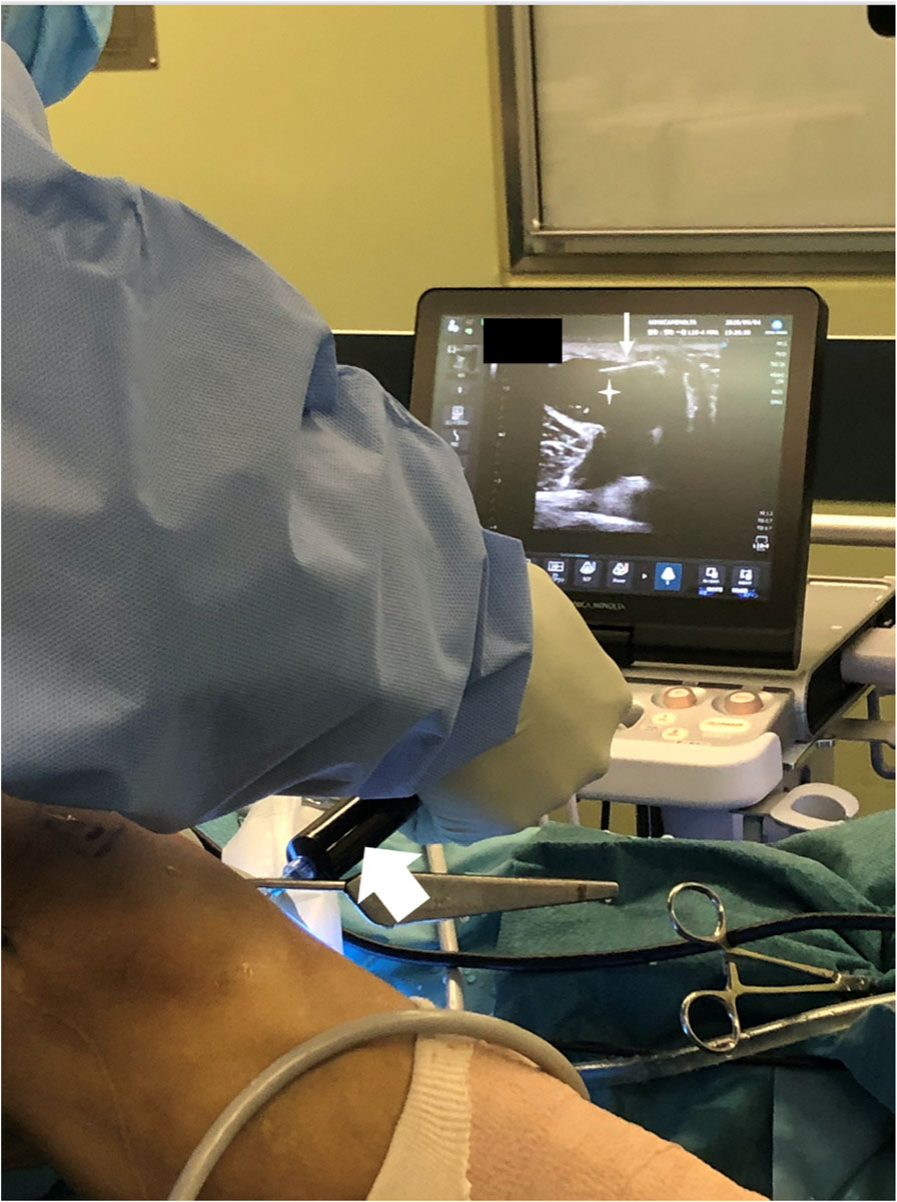
A contrast dye (indigo carmine, broad white arrow) is injected into the popliteal cyst (star) percutaneously using ultrasonography. The 23-gauge needle (narrow white arrow) is clearly visible on the ultrasound screen

**Fig. 4:**
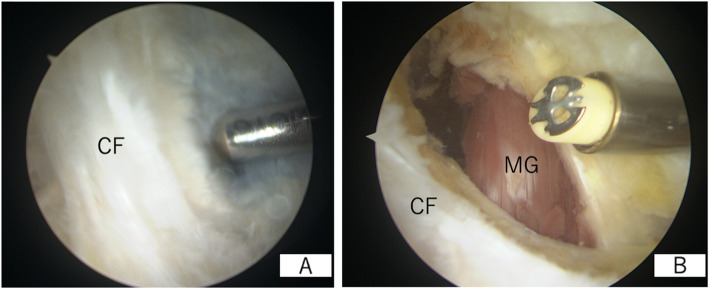
Arthroscopic findings of the posteromedial compartment of the right knee via the anterolateral portal. **a** Indigo carmine is leaking out from the communication channel. **b** The arthroscopic enlargement is completed. CF: capsular fold; MG: medial gastrocnemius

A standard 2-portal approach is used for routine observation; if there is a lesion in the joint that needs to be treated, this is performed. Consequently, a camera is inserted into the posteromedial compartment from the anterolateral portal through the intercondylar notch, and a posteromedial portal is created. After creating the posteromedial portal, the shaver or radiofrequency ablation device is left in place in the joint. In this situation, the back of the knee is subjected to ultrasound, and the needle is inserted into the cyst under ultrasound guidance (Fig. 3). Subsequently, a contrast dye (approximately 10 mL of 0.4% indigo carmine) is injected into the popliteal cyst percutaneously; either the indigo carmine dye leaks out of the communication channel or, if not, the medial margin of the medial gastrocnemius is easily identified since the cyst dilates, making the fold more visible (Fig. 4a). The valve between the semimembranosus and medial gastrocnemius is enlarged using a shaver and radiofrequency ablation. Additionally, the goal of our arthroscopic surgery is to enlarge the communication channel to at least 6 cm long and 3 cm wide (Fig. 4b). Additional resection of the gastrocnemius medialis is never performed. Inner wall resection, called cystectomy, is not performed in any case. After the suction of the irrigation fluid, ultrasonography is immediately performed to confirm the disappearance of the inner cyst fluid (Fig. 2c), and a periarticular multimodal drug is injected under ultrasonography into the septum and inner cyst wall (Fig. 2d). Full knee range of motion and full weight-bearing are allowed from postoperative day 1.

## Discussion

There are various treatment options available for popliteal cysts. While various studies have reported that conservative management of popliteal cysts results in a high rate of cyst persistence [12, 22], ultrasound-guided percutaneous treatment (ultrasound-guided puncture, aspiration, and corticosteroid injection) is one of the few acceptable treatment options, with a recurrence rate of 12.7%. We prefer this conservative treatment as an initial strategy. Historically, open surgery has been widely performed; however, the recurrence rate has been high, ranging from 42% to 63% [6, 8, 24]. In addition, there are concerns about cosmetic issues, wound pain, and wound complications due to the larger wound size.

A recent review reported that the arthroscopic management of intra-articular lesions and enlargement of the communication between the cyst and the articular cavity are effective strategies for popliteal cyst management [30]. Several studies have reported that arthroscopic surgery could allow the identification of the valve and communication enlargement [2, 23] without resection of the posterior horn of the medial meniscus, as first reported by Sansone et al. [28]. Moreover, the success rate of these arthroscopic enlargement procedures is better than that of arthroscopic closure surgeries (80 to 86.4%) [5, 15, 30] and simple open resection (37%) [25], reaching rates of 90 to 100% [2, 7, 17, 20, 23, 28] (Table 1). A meta-analysis by Zhou et al. [30] showed that the success rate of communication enlargement surgery was 96.7% regardless of the resection of the cyst wall, suggesting that it is an ideal strategy for popliteal cyst management.

**Table 1:** Success rates of communication enlargement surgery with and without cystectomy

	Number	Success rate	Cystectomy
Cho et al. 2012 [7]	111	100%	+
Lie and Ng 2011 [20]	10	100%	+
Ahn et al. 2010 [2]	31	96.8%	+
Sansone and Deponti 1999 [28]	30	96.7%	–
Ohishi et al. 2015 [23]	29	93.1%	–
Ji et al. 2009 [17]	44	90.9%	–

The therapeutic benefit of inner wall resection, also described as cystectomy, remains controversial. In a review by Zhou et al. [30], the pooled success rate was 98.2% in the inner wall resection group, compared to 94.7% in the no resection group. However, the difference of approximately 3% was not statistically significant. Furthermore, there was a concern that an additional inner wall resection might increase complication rates. On the contrary, they similarly concluded that there may be a difference in long-term outcomes between the two groups. In a more recent study, Han et al. [12] compared clinical outcomes and complication rates between patients with and without cyst wall resection. Their meta-analysis showed that the recurrence rate was 0% and 6.4% in the cystectomy and the no-cystectomy groups, respectively. However, neither procedure showed an evident superiority over the other since arthroscopic cystectomy was associated with a relatively high incidence of complications. Based on these results, we did not resect the inner wall of the cyst; rather, we focused on enlarging the communication valve.

It remains unknown how large the posteromedial capsule should be resected. Previously, we had performed this procedure without an ultrasound and experienced a patient with recurrence who had undergone an enlargement of 4 cm in length and 2 cm in width. After this experience, we lengthened and widened the enlargement. There is no specific scientific evidence regarding the accurate resection size. Therefore, we enlarged the communication channel to at least 6 cm in length and 3 cm in width during the surgery. After this change, we have not experienced any cases of recurrence so far.

In adults, popliteal cysts are commonly associated with intra-articular disease, resulting in persistent and excessive synovial fluid production [9, 18]. Thus, several studies have demonstrated that the treatment of intra-articular lesions is equally important, and injuries to the medial meniscus and articular cartilage were the most common joint lesions [9, 18, 27]. However, in 16 cases wherein only the intra-articular lesions were treated, favorable therapeutic efficacy was achieved in only 5 patients [26]. Therefore, this suggests that a more important aspect of surgery is to enlarge the communication channels [30]. Nevertheless, symptomatic meniscal or cartilage lesions that are identified arthroscopically at initial routine observation should be treated as needed [2, 30].

Ultrasound-guided surgery has 3 advantages. First, it makes the injection of a contrast dye into the cyst more reliable while allowing for simultaneous accurate identification of the cyst. Similarly, it avoids the risk of neurovascular injury due to erroneous punctures, which is useful in terms of safety. Second, it is possible to evaluate the insides of the cyst immediately after the communication enlargement procedures. Complex cysts are characterized by septation [19], and the necessity of additional procedures, such as resection of the septum, can be determined intraoperatively. Finally, ultrasound-guided multimodal drug injection can supposedly provide a high degree of pain relief. In fact, many of our patients have achieved unassisted gait on the following day without postoperative pain.

Only two patients had undergone this new surgical technique with extremely short-term outcomes (4 and 2 months postoperatively). Although both patients have not shown any symptoms or signs of recurrence, the evaluation of more patients with a longer follow-up period is needed to report our clinical outcomes. However, according to previous literature, it is clear that arthroscopic enlargement surgery can obtain good clinical outcomes with a low recurrence rate [2, 7, 17, 20, 23, 28]. Therefore, we would like to focus our study on this new ultrasound-guided surgical technique for the treatment of popliteal cysts.

## Conclusion

Ultrasound-guided arthroscopic communication enlargement surgery for popliteal cysts can ensure procedure reproducibility with low recurrence rates and be effective for postoperative pain relief. Thus, this combined technique may be an optimal treatment for popliteal cysts.

## Data Availability

Data sharing is not applicable to this article, as no datasets were generated or analyzed during the current study.
